# A micro-/nano-chip and quantum dots-based 3D cytosensor for quantitative analysis of circulating tumor cells

**DOI:** 10.1186/s12951-018-0390-x

**Published:** 2018-09-11

**Authors:** Xuan Wu, Tingyu Xiao, Zhang Luo, Rongxiang He, Yiping Cao, Zhenzhong Guo, Weiying Zhang, Yong Chen

**Affiliations:** 1 Institute for Interdisciplinary Research, Key Laboratory of Optoelectronic Chemical Materials and Devices of Ministry of Education, Jianghan University, Wuhan, 430056 People’s Republic of China; 2 Hubei Province Key Laboratory of Occupational Hazard Identification and Control, Medical College, Wuhan University of Science and Technology, Wuhan, 430065 People’s Republic of China; 3 CNRS-ENS-UPMC, UMR 8640, Ecole Normale Supérieure, 24 Rue Lhomond, 75005 Paris, France

**Keywords:** Micropillar, Nanofiber, Quantum dots, Three-dimensional cytosensor, Circulating tumor cells

## Abstract

**Background:**

Due to the high transfer ability of cancer cell, cancer has been regarded as a world-wide high mortality disease. Quantitative analysis of circulating tumor cells (CTCs) can provide some valuable clinical information that is particularly critical for cancer diagnosis and treatment. Along with the rapid development of micro-/nano-fabrication technique, the three-dimensional (3D) bionic interface-based analysis method has become a hot research topic in the area of nanotechnology and life science. Micro-/nano-structure-based devices have been identified as being one of the easiest and most effective techniques for CTCs capture applications.

**Methods:**

We demonstrated an electrospun nanofibers-deposited nickel (Ni) micropillars-based cytosensor for electrochemical detection of CTCs. Breast cancer cell line with rich EpCAM expression (MCF7) were selected as model CTCs. The ultra-long poly (lactic-co-glycolic acid) (PLGA) nanofibers were firstly-crosswise stacked onto the surface of Ni micropillars by electrospinning to construct a 3D bionic interface for capturing EpCAM-expressing CTCs, following immuno-recognition with quantum dots functionalized anti-EpCAM antibody (QDs-Ab) and forming immunocomplexes on the micro-/nano-chip.

**Results:**

The Ni micropillars in the longitudinal direction not only play a certain electrical conductivity in the electrochemical detection, but also its special structure improves the efficiency of cell capture. The cross-aligned nanofibers could simulate the extracellular matrix to provide a good microenvironment which is better for cell adhesion and physiological functions. Bioprobe containing quantum dots will release Cd^2+^ in the process of acid dissolution, resulting in a change in current. Beneath favourable conditions, the suggested 3D cytosensor demonstrated high sensitivity with a broad range of 10^1^–10^5^ cells mL^−1^ and a detection limit of 8 cells mL^−1^.

**Conclusions:**

We constructed a novel 3D electrochemical cytosensor based on Ni micropillars, PLGA electrospun nanofibers and quantum dots bioprobe, which could be used to highly sensitive and selective analysis of CTCs. More significantly, the 3D cytosensor can efficiently identify CTCs from whole blood, which suggested the potential applications of our technique for the clinical diagnosis and therapeutic monitoring of cancers.

## Background

Due to the high transfer ability of cancer cell, cancer has been regarded as a world-wide high mortality disease. It is reported that the increase amount of circulating tumor cells (CTCs) emerged in the peripheral blood will result in the cancer metastasis and relapse [1–3]. Quantitative analysis of CTCs can provide some valuable clinical information that is particularly critical for cancer diagnosis and treatment. However, the number of CTCs in the whole bloodstream is usually very low (a few to hundreds per milliliter), quantification of CTCs to assess cancer metastasis faces a huge challenge [4, 5]. In the past few years, various approaches have already been presented to enrich/count CTCs, including flow cytometry [6], immunemagnetic beads [7], microfluidic devices [8–10] and so on. Though those developed methods have been greatly accepted, their sensitivity is still a major flaw. Therefore, developing a highly sensitive CTCs assay method is urgent for predicting cancer metastasis and relapse.

Along with the rapid development of micro-/nano-fabrication technique, the three-dimensional (3D) bionic interface-based analysis method has become a hot research topic in the area of nanotechnology and life science. 3D bionic interface, usually displayed in the form of micro-/nano-structure (e.g., micropillars [11], nanofibers [12], nanotubes [13, 14] and nanopillars [15]), are endowed with good biocompatibility and large specific surface area [16–19]. Simultaneously, combined with the nanoscale characters implanted in cellular surface elements (e.g., microvilli and filopodia) and extracellular matrix (ECM) scaffolds, 3D bionic interface provides a comfortable microenvironment where cell capture and rare cell detection could be achieved. Furthermore, functional nanomaterials have been introduced into 3D bionic interface, significantly diversifying the detection methods and enhancing the detection sensitivity. Wang’s group has ever reported a novel silicon nanopillar coated with anti-EpCAM-based biosensing platform for CTCs capture and subsequent sensitive assay [20]. Our group also developed a graphene-modified 3D microchip-based supersandwich cytosensor for quantitative immunoassay of CTCs [21].

Nanostructure-based devices have been identified as being one of the easiest and most effective techniques for CTC capture applications. Electrospinning is an easy and universal nanofabrication technique, by which a variety of soluble and fusible polymers could be transferred to form the desired nanofibers with steerable diameters from a few nanometers to several micrometers [20, 22–24]. The prepared nanofibers are coated onto the 3D bionic interface to simulate an excellent porous microenvironment, which is especially beneficial for cellular filopodia climbing, helping cell adhesion and growth.

Herein, we demonstrated an electrospun nanofibers-deposited nickel (Ni) micropillars-based cytosensor for electrochemical detection of CTCs. Breast cancer cell line with rich EpCAM expression (MCF7) were selected as model CTCs. The ultra-long poly (lactic-co-glycolic acid) (PLGA) nanofibers were firstly-crosswise stacked onto the surface of Ni micropillars by electrospinning to construct a 3D bionic interface for capturing EpCAM-expressing CTCs, following immuno-recognition with quantum dots functionalized anti-EpCAM antibody (QDs-Ab) and forming immunocomplexes on the micro-/nano-chip. The signal current response was achieved by electrochemical assay of the released cadmium ion (Cd^2+^) after acid-dissolving QDs from immunocomplexes. Using this 3D substrate, we dependably gathered cancer cells from synthetic CTC blood samples. The integration of crossed PLGA nanofibers and conductive Ni micropillars not only provide an excellent microenvironment for CTCs capture, preventing CTCs from flowing away in the process of rinse and increasing the capture efficiency of target cell, but also greatly amplify the current signal, improving detection sensitivity. Coupling with 3D micro-/nano-structure, the proposed biosensing platform exhibited great potential for on-site monitoring cancer progress. We expect that this platform could be applied in isolating rare populations of cells that cannot be easily realized using existing technologies, as well as in early diagnosis and longitudinal monitoring of cancer in the clinic.

## Methods

### Chemicals

Indium tin oxide (ITO) glasses with a resistance of 10 Ω were purchased from South of China Xiangcheng Technology. AZ9260 photoresists and the developer AZ-300MIF were purchased from AZ Electronic Materials Corp. (Philadelphia, PA). Bovine serum albumin (BSA), fluorescein diacetate (FDA) and streptavidin (SA) were purchased from Sigma (St. Louis, MO). A quantum dots (Qdot) 585 CdSe@ZnS antibody labeling kit was purchased from Life Technologies. DMEM medium for cell culture was obtained from GIBCO. Biotinylated goat IgG polyclonal anti-EpCAM antibody was obtained from R&D Systems (Minneapolis, MN). Pan-Cytokeratin antibody (C11) Alexa Fluor^®^ 488 and CD45 Antibody (2D-1) PE were purchased from Santa Cruz Biotechnology. Cancer patient serum was provided by Zhongnan Hospital of Wuhan University (Wuhan, China). All other chemicals used in this study were analytical-grade. All solutions were prepared with ultrapure water obtained from a Millipore water-purification system (Millipore, USA).

### Cell culture

The MCF7 cells were cultured in DMEM, which was supplemented with 10% fetal bovine serum (FBS) and 100 μg/mL penicillin–streptomycin in an incubator (5% CO_2_, 37 °C). Jurkat cells were cultured in RPMI Medium 1640 supplemented with 10% fetal bovine serum. After the concentration of cells reached 1 × 10^5^ cells mL^−1^, the cells were collected by centrifugation at 1000 rpm for 3 min respectively.

### Fabrication of the 3D Ni micropillars

**Scheme 1:**
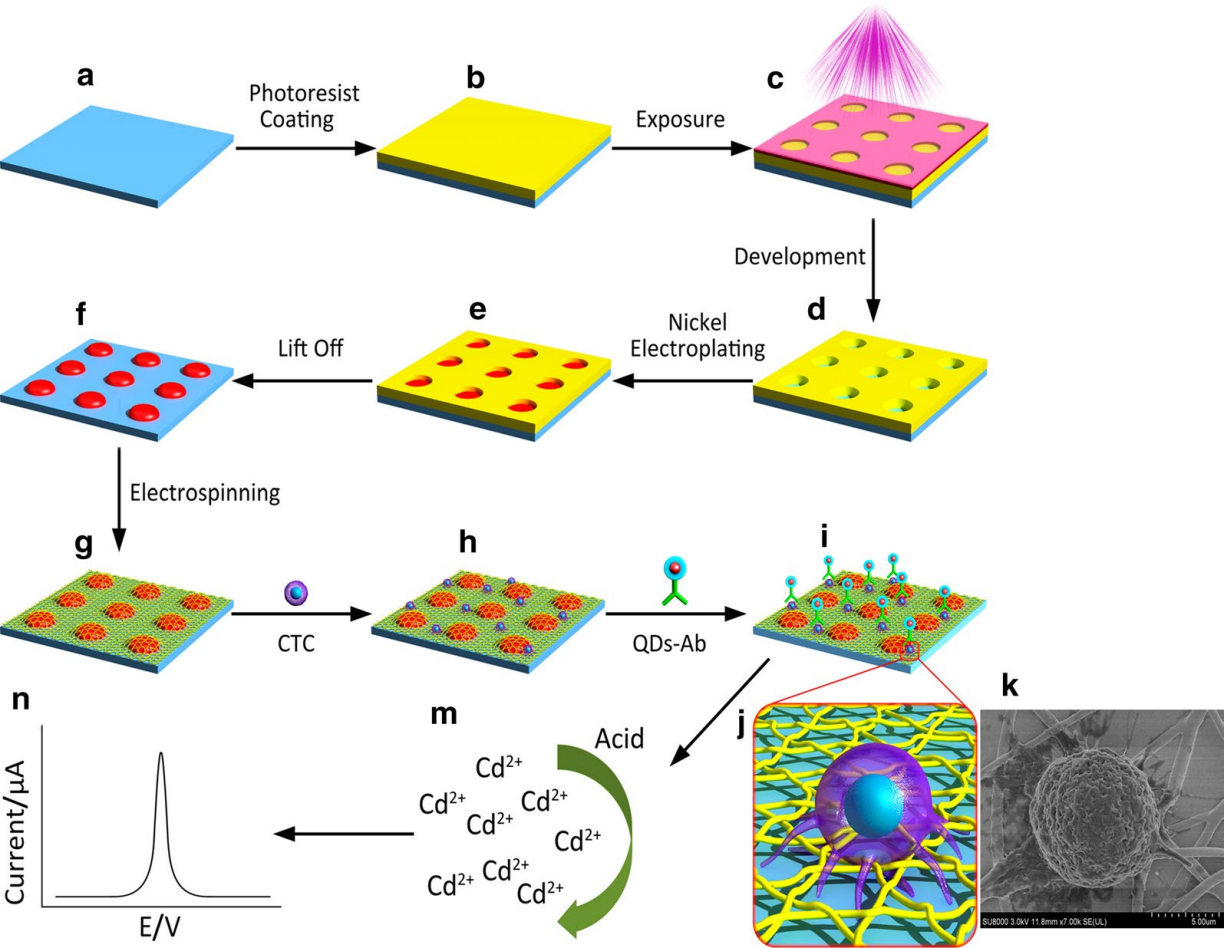
Schematic representation of the integrated fabrication of 3D cytosensor and electrochemical detection of CTCs. **a**–**f** Fabrication procedure of the Ni micropillars on the ITO glass. **g** Fabrication of electrospun PLGA nanofibers on Ni micropillars. **h** CTCs were captured and **i** QDs-anti-EpCAM antibody conjugates were recognized. **j** The amplification of captured CTC and **k** SEM image of CTC in microenvironment. **m**, **n** Principles of electrochemical assay

Scheme 1a–f shows the fabrication procedure of the Ni micropillars on the ITO glass. AZ9260 photoresist was firstly spin-coated onto the ITO glass slide, which was then washed with ultrasonic rinsing in ethanol and distilled water. After exposure with a Chromium mask, which was prepared by Laser Lithography System (Heidelberg, μPG501), beneath UV light, the photoresist film was processed in AZ developer (1:3 v/v AZ-300 MIF/H_2_O) for 1 min to obtain a desired pattern. Subsequently, the ITO glass slide was immersed in a Ni electroplating solution with the bulk of Ni block as an anode. After electroplating with the current density of 0.05 A/cm^2^ for 5 min at 50 °C, 3D Ni micropillars with 10 μm height were obtained on the ITO glass slide. Finally, the remaining photoresist was lifted off in acetone.

### Fabrication of electrospinning PLGA nanofibers

Electrospinning PLGA nanofibers were fabricated as follows: PLGA powder (2 g) was liquefied in a mixed solvent of DMF-THF (15 mL, v/v = 3:1) with overnight stirring up to the point when a transparent and homogeneous solution was acquired. Recently assembled PLGA solution filled a stainless steel needle (inner diameter = 0.6 mm) that was connected to a high-voltage DC power supply (Wuhan, China). The electrospinning nanofibers were obtained by using an electrospinning apparatus at a feeding rate of 0.6 mL/h, an electric voltage of 12 kV, and a tip to collector distance of 15 cm. Nanofibers were directly electrospun onto 3D Ni micropillars. The formed 3D micro-/nano-structures were dried in a vacuum oven over 24 h to remove the residual organic solvent and moisture. Finally, Au nanoparticle was plated to the resulted substrate by the ion sputter coater to obtain modified electrode.

### Preparation of QDs-anti-EpCAM antibody conjugates

A Qdot 585 antibody labeling kit was utilized to conjugate the anti-EpCAM antibody to QDs. Prior to conjugation, anti-EpCAM was decontaminated via a gel filtration column (Superose 12, Pharmacia-LKB) to eliminate surfactants and other proteins, including BSA. The concentration of anti-EpCAM was about 0.5 mg/mL. Conjugation was performed according to the manufacturer’s procedure. The obtained QDs-anti-EpCAM conjugate was stored at 4 °C for further use. The conjugate concentration was established (1.5 × 10^6^ M) by quantifying the absorbance density at 585 nm with an Ultrospec 2100 Pro UV/visible spectrophotometer.

### Quantitative detection MCF7 cells with 3D cytosensor

The MCF7 cell suspension (100 μL) was introduced onto the 3D Ni/PLGA micro-/nano-chip and kept in an incubator (5% CO_2_, 37 °C) for 1 h. Then, the device was incubated with QDs-anti-EpCAM (100 μL) at 37 °C for 1 h. After rinsing with PBS, a portion of HCl (10 μL, 1 M) was dropped on the chip to dissolve the captured QDs. The detection solution with 10 μg/mL Hg in acetate buffer (0.2 M, pH 4.6) was then added for quantitative measurement with a CHI 660E electrochemical workstation. As a control, substrates only with micropillars or PLGA nanofibers were also examined in parallel.

## Results and discussion

### Efficient capture of CTCs with the 3D Ni/PLGA micro-/nano-chip

**Fig. 1:**
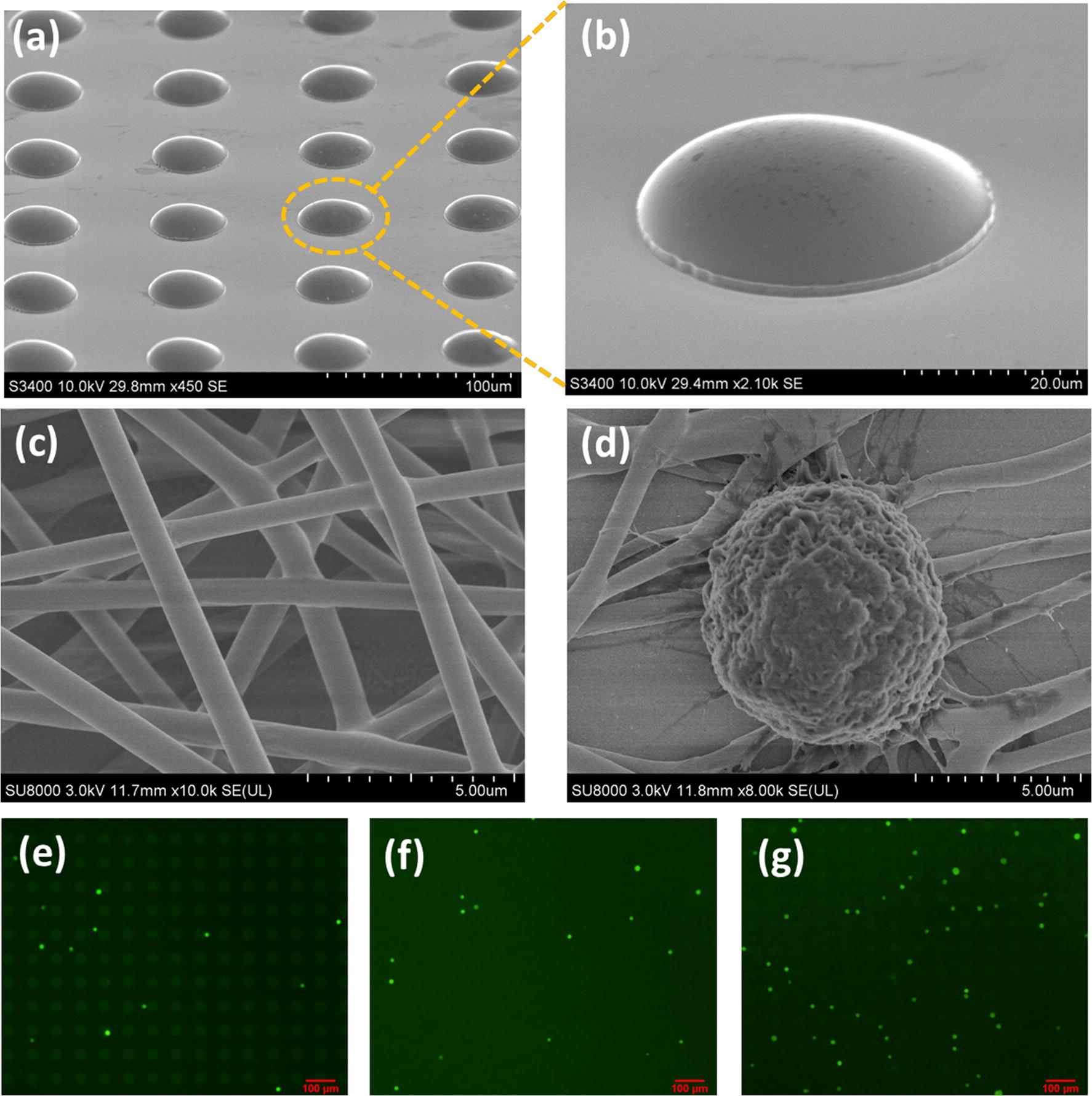
The 3D Ni/PLGA micro-/nano-chip local topographic interactions among the micropillars, nanofibers and CTCs. The SEM images of **a**, **b** Ni micropillars, **c** PLGA nanofibers and **d** MCF7 cells were captured on 3D Ni/PLGA micro-/nano-chip. FDA fluorescence micrographs of target cells were captured on **e** Ni micropillars substrate, **f** PLGA nanofibers substrate and **g** 3D Ni/PLGA patterned substrate

The 3D Ni/PLGA micro-/nano-chip was prepared as illustrated in Scheme 1. As can be seen from Fig. 1a, b, the micropillars were fabricated with the diameter of 40 μm and the height of 10 μm. The diameter of PLGA nanofibers was about 500 nm (Fig. 1c). To test the cell-capture performance of the 3D Ni/PLGA micro-/nano-chip, we fabricated substrates only with Ni micropillars, only with PLGA nanofibers and both with Ni/PLGA, respectively, to compare their cell affinity. A cell suspension (10^5^ cells mL^−1^) was introduced onto the chips, and then incubated at 5% CO_2_ and 37 °C for 1 h. As shown in Fig. 1e–g, the Ni/PLGA micro-/nano-chip captured much more cells, while the Ni micropillars surface and PLGA nanofibers surface captured few ones. More important, MCF7 with fully outspread pseudopodia connected to the 3D Ni/PLGA surface could be clearly observed (Fig. 1d). It suggests that the synergistic interplay between Ni and nanofibers could possibly be held accountable for the improved cell-capture yields. This effect likely due to the 3D Ni/PLGA micro-/nano-chip enhanced local topographic interactions among the micropillars, nanofibers and cells.

**Fig. 2:**
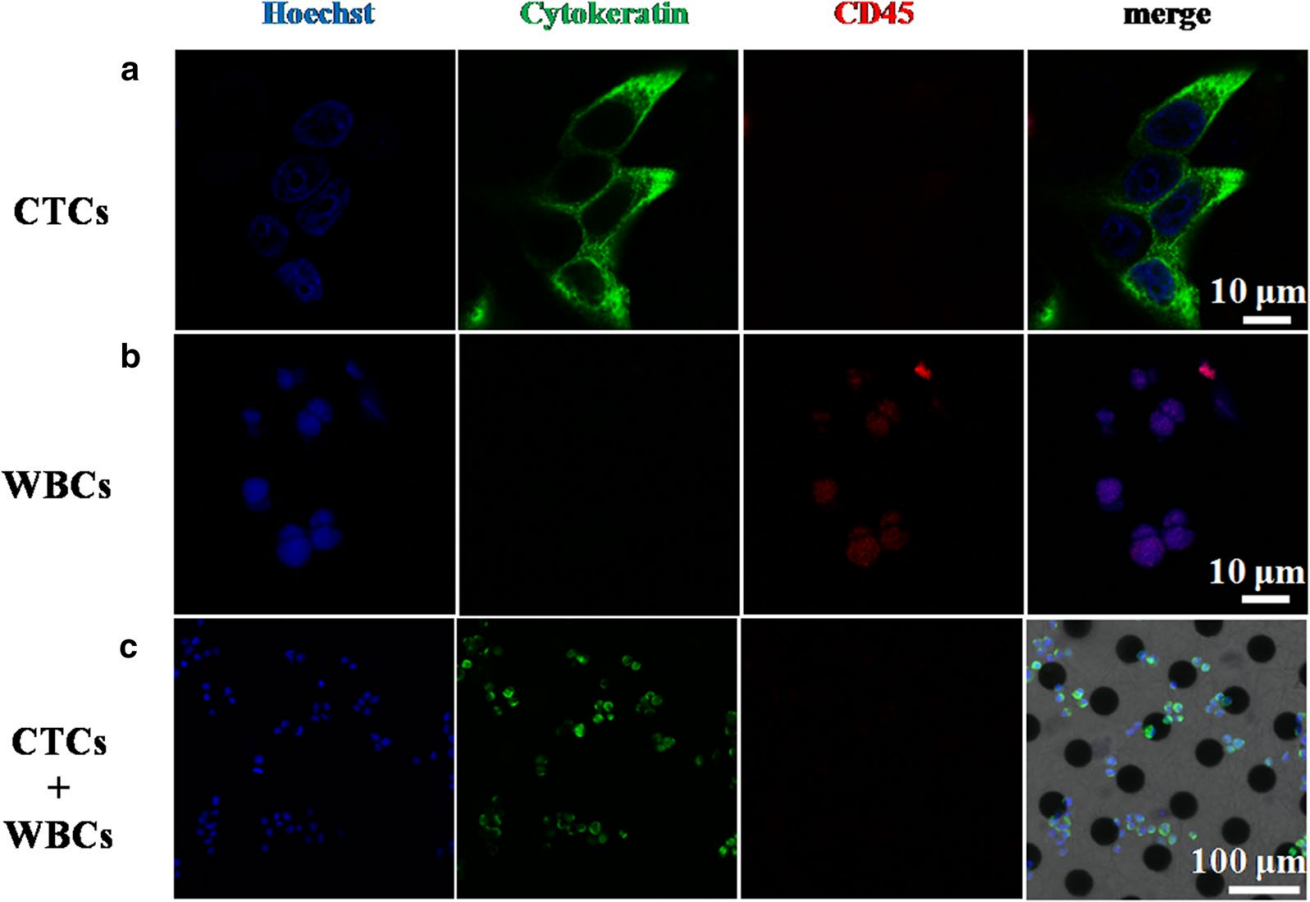
The three-color immunocytochemistry technique with Alexa Fluor^®^ 488-labeled anti-CK, PE-labeled anti-CD45 and Hoechst nuclear staining was used to distinguish **a** CTCs from **b** non-specifically trapped WBCs on the 3D micro-/nano-chip. **c** Isolation of CTCs from mixed cell suspension on 3D bionic interface

To differentiate captured CTCs and white blood cells (WBCs), as shown in Fig. 2a–h, three-color immunostaining was performed using Alexa Fluor^®^ 488-labeled anti-CK (Cytokeratin, a protein marker for epithelial cells) and PE-labeled anti-CD45 (a marker for WBCs) as well as Hoechst nuclear staining. Following image capture, the integrated information was used to distinguish CTCs (Hoechst+/CK+/CD45−, cell size > 10 μm) (Fig. 2a) from WBCs (Hoechst+/CK−/CD45+, cell size < 10 μm) (Fig. 2b). On this basis, the mixed suspension consists of CTCs and WBCs were added onto the 3D Ni/PLGA micro-/nano-chip at the same concentration of 10^5^ cells mL^−1^ and incubated at 37 °C, 5% CO_2_ for 1 h. After 1× PBS rinse and immunostaining with anti-CK and anti-CD45, it could be observed from Fig. 2c that only CTCs were captured on the 3D substrate. It is mostly due to the size effect that the diameters of the pseudopodia and the PLGA nanofibers are well suited to acquire enough contact and effective adhesive force, causing increased cell/substrate affinity.

### Optimization of experimental conditions

**Fig. 3:**
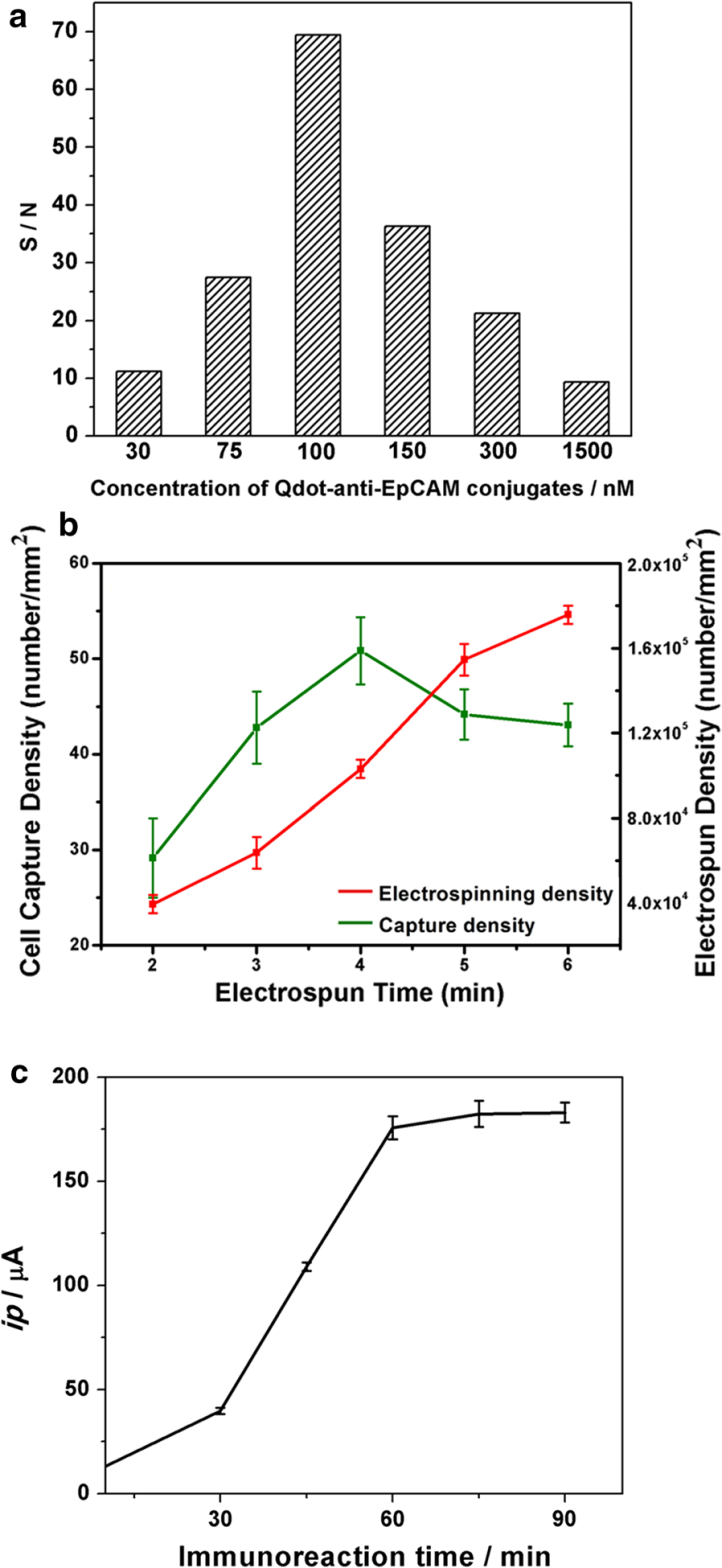
Optimization of parameters of the 3D cytosensor. **a** Qdot-anti-EpCAM conjugates concentration on the signal-to-noise (S/N) ratio of cytosensor. **b** The relationship among the electrospinning time, the PLGA nanofiber density and MCF7 capture density. **c** Immunoreaction time on the signal responses of cytosensor. They were performed by applying a 10^5^ cell mL^−1^ to the substrate

The electrochemical activity of the cytosensor is impacted by several parameters, including the portion of QD-anti-EpCAM conjugate attached to the MCF7 cells on the surface of the 3D Ni/PLGA micro-/nano-chip. It directly affects the electrochemical response of the cytosensor since the signal mainly depends on the amount of QD-anti-EpCAM conjugates recognized by CTCs. The surplus of Qdot conjugates results in increasingly nonspecific adsorption. To acquire a maximum reaction and a minimum nonspecific adsorption, the optimal amount of QD-anti-EpCAM was determined. We diluted the original conjugate solution to various concentrations and investigated the influence on the signal-to-noise (S/N) ratio of the cytosensor with 10^5^ cell mL^−1^. PBS acted as a control. As shown in Fig. 3a, the S/N ratio was discovered to be the highest for dispensing 100 nM QD-anti-EpCAM. Nevertheless, the reduction of the S/N ratio at a concentration > 100 nM is because of the rise of the background signal causing surplus portions of Qdot conjugates while at a lower concentration, the reduction of the S/N ratio is attributed to the lowering of the signal due to low Qdot-anti-EpCAM availability. Consequently, 100 nM Qdot-anti-EpCAM was typically utilized as the optimal concentration during the whole study.

In addition, in order to investigate how PLGA nanofiber density affects the cell capture density, we performed cell-capture experiments with MCF7 cells on a series of the alternately 3D Ni/PLGA micro-/nano-substrate (PLGA nanofiber with electrospun time of 2, 3, 4, 5, 6 min). As shown in Fig. 3b, the cell capture density increased with increasing electrospun time from 2 to 4 min. When the electrospun time was beyond 4 min, the cell capture densities were reduced, which is likely since too packed nanofiber limited the cell capture. These results reveal that the local topographic interactions are correlated with the characteristics of the PLGA nanofiber.

The incubation time between CTCs and QDs-Ab conjugates has also been explored by electrochemical response, as shown in Fig. 3c. The current reaction was elevated with the rising immunoreaction time, and it was inclined to be stable after about 60 min. Thus, 60 min was selected as the optimized reaction time.

### Electrochemical detection of CTCs

**Fig. 4:**
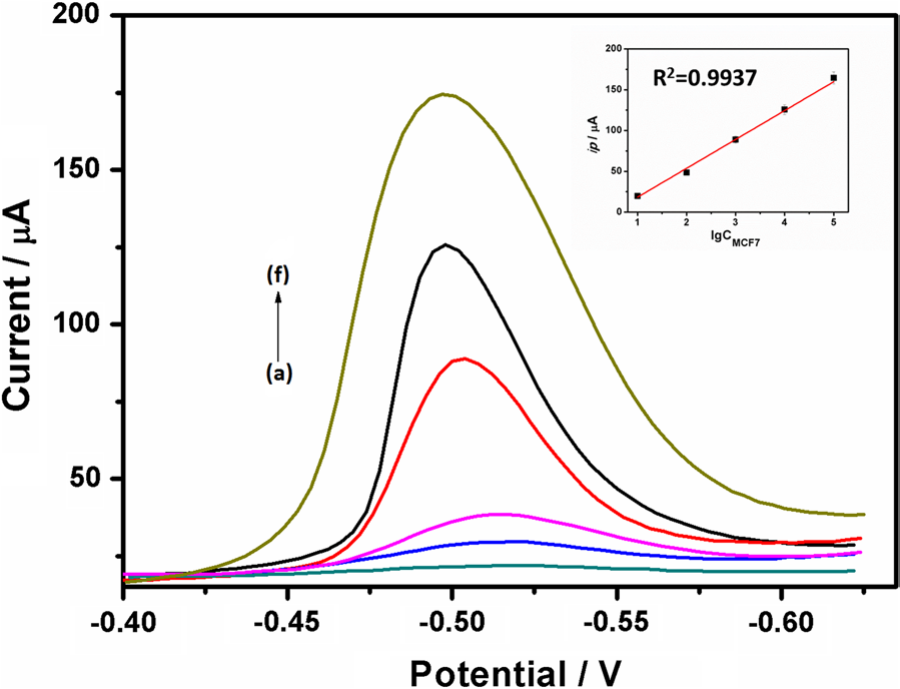
Differential pulse voltammetry responses of the supersandwich cytosensor incubated with (a) Jurkat cells at 10^5^ cells mL^−1^ and (b–f) different concentrations of MCF7 cells: 10^1^, 10^2^, 10^3^, 10^4^, 10^5^ cells mL^−1^. Insert: Calibration curve of MCF7

Under the optimal experiment condition, the proposed supersandwich cytosensor was challenged to test different concentrations of MCF7 cells. As depicted in Fig. 4, the electrochemical response increased with the amount of MCF7 cells increasing, showing a good linear range of 10^1^–10^5^ cells mL^−1^ with a correlation coefficient of 0.9937 (n = 3). The detection limit at a signal-to-noise ratio of 3σ—where σ is the standard deviation of the signal in a blank solution—declined to 8 cells mL^−1^, which greatly benefited from the large specific surface area of electrospun PLGA nanofiber, excellent conductivity of Ni micropillar and signal amplification of QDs. The comparison of the study about CTCs detection methods was summarized in Table 1.

**Table 1:** Comparison of the sensitivity of different CTCs detection methods

Methods	Linear range (cells mL^−1^)	Detection limit (cells mL^−1^)	Refs
3D cytosensor	10^1^–10^5^	8	Present work
SERS nanoprobes	1–10^2^	1	[25]
SERS nanoparticles	5–500	5	[26]
GASI chip	1–51	1	[27]
Microchip cytosensor	10^1^–10^7^	10	[21]
LSAW aptasensor	10^2^–10^7^	32	[28]
PEC biosensor	10^2^–10^6^	58	[29]
Colorimetric aptasensor	10^2^–10^4^	40	[30]
Aptamer/QDs cytosensor	10^2^–10^6^	50	[31]

**Fig. 5:**
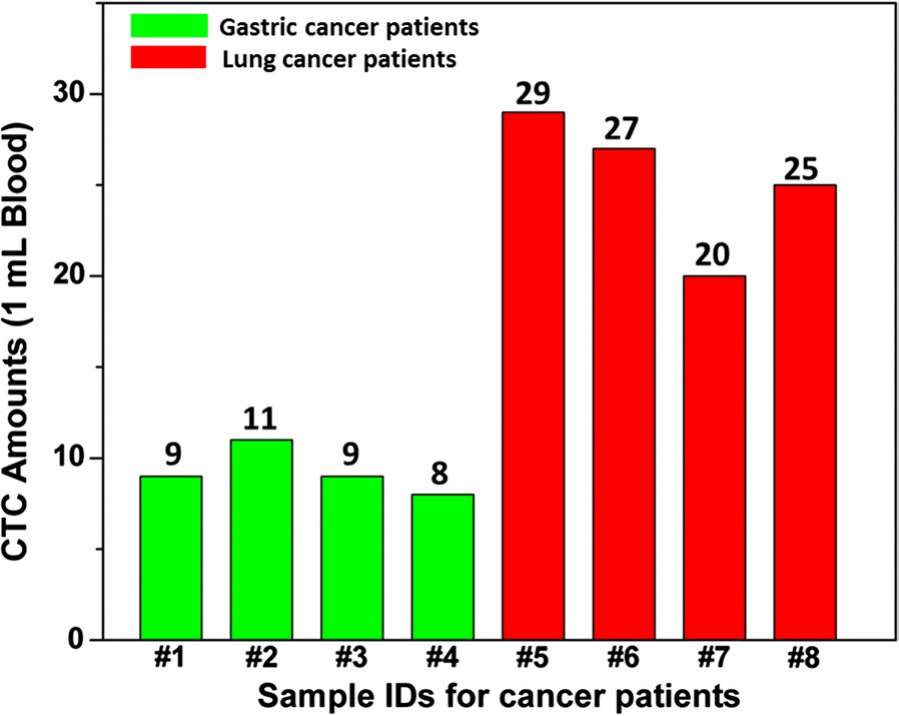
Detection of CTC amounts in the 1 mL blood samples collected from cancer patients. Green columns: gastric cancer patients. Red columns: lung cancer patients. The amounts were calculated from the calibration curve

A series of human plasma samples were further used to test the accuracy and viability of the proposed approach. These samples were established by spiking various amounts of MCF7 to human plasma. The results were summarized in Table 2, which showed the recoveries are in the range of 93.5–105%, indicating that the constructed device provides a novel technique for fast, selective, and sensitive detection of MCF7 in actual specimens. In addition, CTC-capture study was performed on peripheral blood samples from gastric and lung cancer patients. The peripheral blood samples were donated by two types of cancer patients with different stages of the disease and preserved in blood collection tubes, and the results were summarized in Fig. 5. Serum sampling was conducted based on ethics principles established by the biosafety committee at Zhongnan Hospital of Wuhan University and each of the donors signed the consent forms.

**Table 2:** Comparison of cytosensor values with known amounts of MCF7 spiked in human plasma

Sample no.	1	2	3	4	5
Know (cell mL^−1^)	20	50	100	200	500
Cytosensor (cell mL^−1^)	21 ± 1	51 ± 2	101 ± 5	187 ± 10	482 ± 19
Recovery (%)	105.0%	102.0%	101.0%	93.5%	96.4%

## Conclusions

In summary, we have tactfully fabricated a cancer cell capture and analysis 3D micro-/nano-chip, which achieves effective capture and sensitive quantitation of CTCs mediated by electrochemical assay. The considerable capture performance benefit from a 3D bionic interface provided by Ni micropilliars and electrospinning PLGA nanofiber net where cancer cells can easily adhere. Meanwhile, a supersensitive analysis method is worked out by electrochemical detection Cd^2+^ released from QDs using an aliquot of HCl. More importantly, using these 3D micro-/nano-chips, we accurately gathered cancer cells from synthetic CTC blood samples and from whole blood samples obtained from gastric and lung cancer patients.

## Abbreviations

CTCs: circulating tumor cells
3D: three-dimensional
Ni: nickel
PLGA: poly (lactic-co-glycolic acid)
QDs: quantum dots
Cd^2+^: cadmium ion
ITO: indium tin oxide
BSA: bovine serum albumin
FDA: fluorescein diacetate
WBCs: white blood cells
S/N: signal-to-noise
